# Pneumomediastinum and Subcutaneous Emphysema: An Unexpected Complication After Noninvasive Ventilation in a COVID-19 Patient

**DOI:** 10.7759/cureus.78379

**Published:** 2025-02-02

**Authors:** Mariana Sousa, Ana de Matos Valadas, Joana Rodrigues dos Santos, Inês Silva, Lígia Peixoto

**Affiliations:** 1 Internal Medicine, Hospital de Santa Maria, Unidade Local de Saúde de Santa Maria, Lisboa, PRT

**Keywords:** alveolar damage, covid-19, pneumomediastinum, pneumonia, subcutaneous emphysema

## Abstract

Pneumomediastinum is a rare clinical condition defined by the presence of free air in the mediastinum. We present the case of a 57-year-old male with severe COVID-19 pneumonia, complicated by pneumomediastinum and subcutaneous emphysema, in a patient without identifiable risk factors for such complications. The patient, a non-smoker with no known lung comorbidities, received medical treatment for COVID-19 and underwent non-invasive ventilation prior to the onset of emphysema. Like most cases, it resolved spontaneously with conservative management, including close monitoring, bed rest, analgesia, and oxygen therapy. The patient was discharged in stable condition.

## Introduction

Pneumomediastinum is a rare clinical condition defined by the presence of free air in the mediastinum. It predominantly affects males, approximately 76%, and the estimated prevalence of pneumomediastinum is 1 in 25,000 among the 5-34 year-old population [1,2]. It can be associated with several structural lung diseases, including chronic obstructive lung disease, asthma, and interstitial lung diseases. Other predisposing factors include tobacco or marijuana smoking, illicit drug use (such as heroin, cocaine, or nitrous oxide), and vigorous Valsalva maneuvers [3-5]. It more often results from blunt trauma or esophageal perforation and is also known to occur as a consequence of barotrauma, including the use of positive airway pressure and mechanical ventilation [1].

The exact prevalence of pneumomediastinum among COVID-19 patients remains unknown [3]. However, the effect the virus has on the lungs, coupled with the need for ventilatory support in severe cases, raises concerns about potential complications, including pneumomediastinum and subcutaneous emphysema [4,5].

In this report, we discuss a case of pneumomediastinum in a patient with severe COVID-19 pneumonia who required non-invasive ventilation, emphasizing the clinical presentation and management strategies.

## Case presentation

A 57-year-old male with a history of gastroesophageal reflux disease (GERD) presented to our emergency department after eight days of fever, cough, anosmia, diarrhea, worsening dyspnea, and fatigue. He had completed five days of antibiotics (amoxicillin/clavulanic acid and azithromycin) before presentation. The patient tested positive for COVID-19 via RT-PCR on day two of symptom onset. He was a non-smoker, did not consume alcohol, and had no history of drug use.

Upon arrival, his oxygen saturation was 86% on room air, improving to 94-95% with 35% FiO2 oxygen supplementation. His respiratory rate was 27 breaths per minute, with a temperature of 38.8°C, pulse of 65 bpm, and blood pressure of 111/71 mmHg. Physical examination revealed no additional abnormalities.

Table 1 reveals the main laboratory findings and Table 2 specifies the relevant results of arterial blood gas analysis on oxygen therapy.

**Table 1:** Patient's laboratory findings at admission.

Laboratory parameters	Value (units)	Reference value
Hemoglobin	15.2 g/dL	12.0-15.3
Leukocytes	9.1x10^9 ^/L	4.0-11.0
Neutrophils	7.6x10^9 ^/L	1.9-7.5
C-reactive protein	17.8 mg/dL	<0.5
Procalcitonin	0.25 ng/mL	<0.5
D-dimer	0.71 ug/mL	0.0-0.5
Lactate dehydrogenase	600 U/L	100-250

**Table 2:** Arterial blood gas analysis under 35% supplemental oxygen therapy.

Laboratory parameters	Value (units)	Reference value
pH	7.44	7.350-7.450
pCO2	29.7 mmHg	35.0-45.0
pO2	70.4 mmHg	75.0-100
HCO3	22.5 mmol/L	22-26
Lactate	8 mg/dL	4.5-18

**Figure 1:**
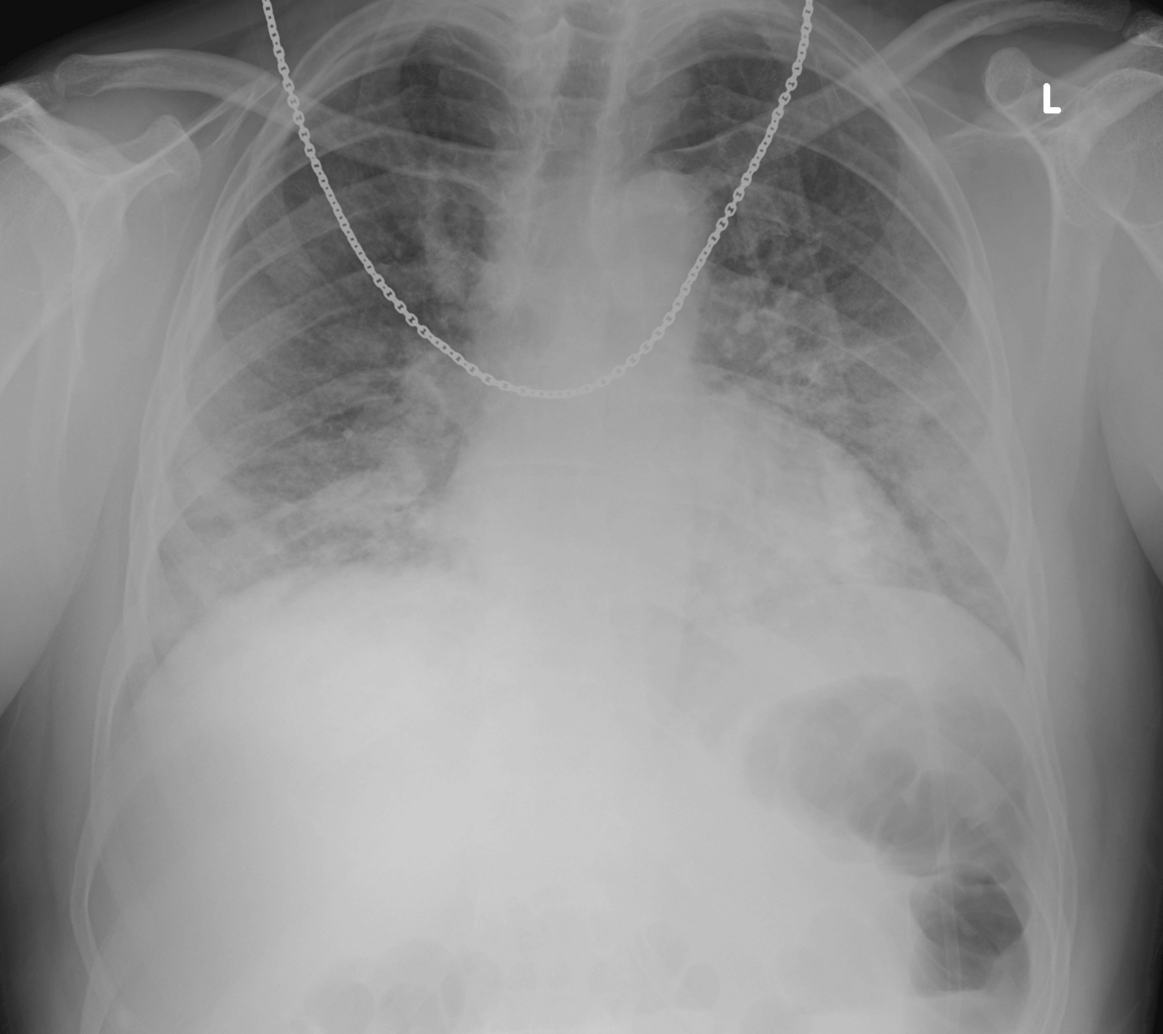
Chest X-ray during initial hospital presentation showing COVID-19 pneumonia.

A chest X-ray showed bilateral bronchovascular prominence, without signs of consolidation (Figure 1).

The chest computed tomography (CT) revealed extensive bilateral peripheral ground-glass opacities affecting approximately 50% of the lung parenchyma, with some areas of focal consolidation and "crazy-paving" patterns suggestive of organizing pneumonia, but no mediastinal abnormalities.

The patient was treated with methylprednisolone, remdesivir, and oxygen via a Venturi mask (40% FiO2). Despite initial management, the patient experienced progressive respiratory decline and was transitioned to high-flow nasal cannula (HFNC) oxygen therapy with 100% FiO2 at 60 L/min. The patient was encouraged to adopt prone positioning as per institutional protocol.

Due to worsening oxygen requirements (P/F ratio of 78) and a chest X-ray showing increased bilateral infiltrates, the patient was transferred to the ICU and started on continuous positive airway pressure (CPAP) therapy at 8 cm H2O with 100% FiO2. A therapeutic dose of enoxaparin was initiated due to elevated D-dimer levels, indicating possible microthrombotic phenomena.

Over the next three days, the patient showed clinical improvement, and his oxygen requirements continued to decrease, allowing progressive reduction of FiO2. He was started on HFNC again, which he utilized for five days. On day 12 of hospitalization, he was transferred to the internal medicine service under a Venturi mask (40% FiO2) and 80 mg/day of methylprednisolone. Remdesivir was suspended on day 4 of treatment because of liver toxicity.

**Figure 2:**
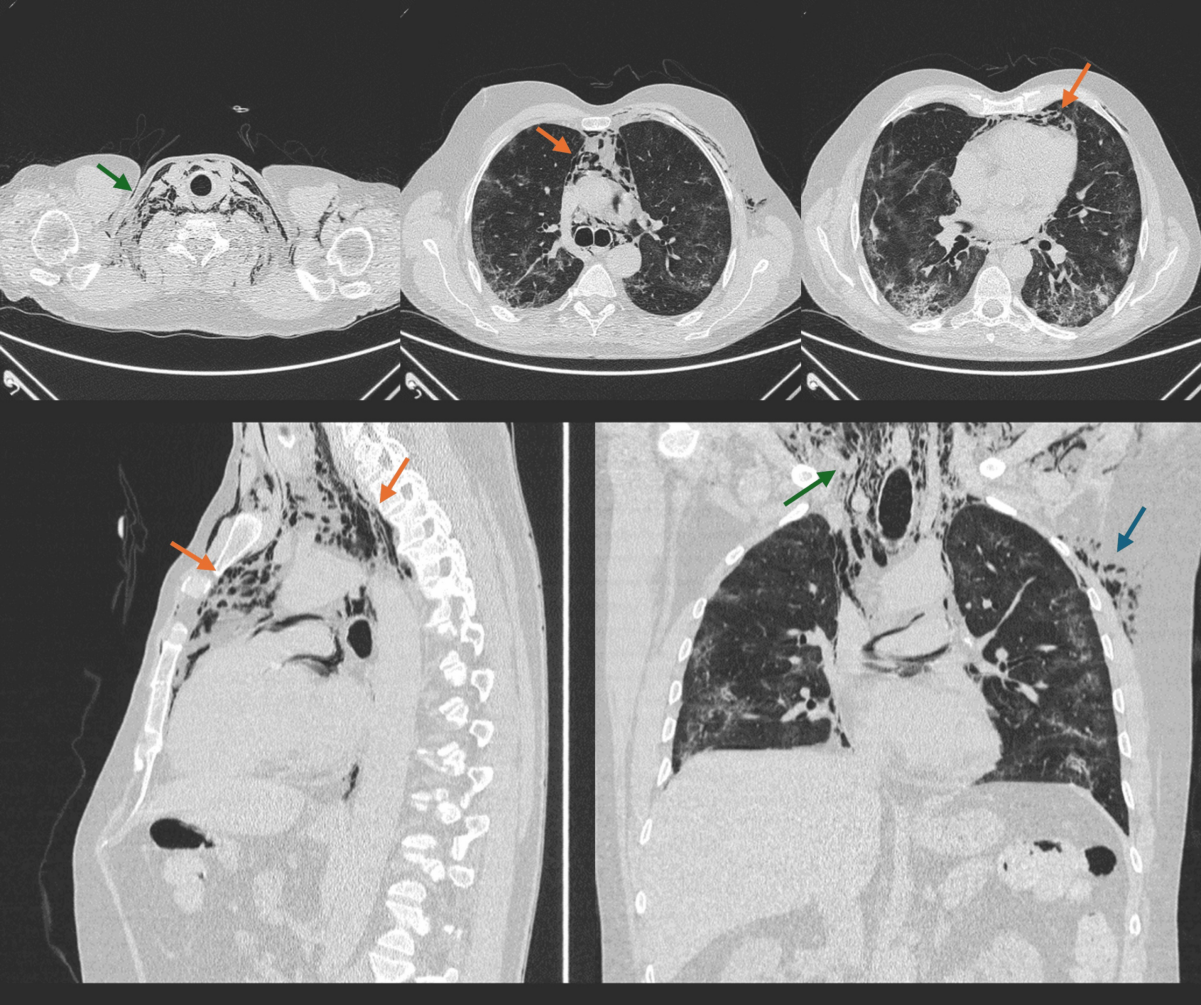
CT scan showing severe pneumomediastinum (orange arrows), and emphysema of the soft tissue of the neck (green arrows) and left lateral chest wall (blue arrow) in a patient with COVID-19 pneumonia.

Over the next four days, the patient developed significant neck swelling with crepitus detected upon examination. A repeat chest and neck CT scan revealed moderate to severe pneumomediastinum extending through various mediastinal compartments, accompanied by subcutaneous emphysema extending into the nasopharynx and soft tissues of the chest wall. The extent of organizing pneumonia had also increased, involving up to 75% of the pulmonary parenchyma (Figure 2).

**Figure 3:**
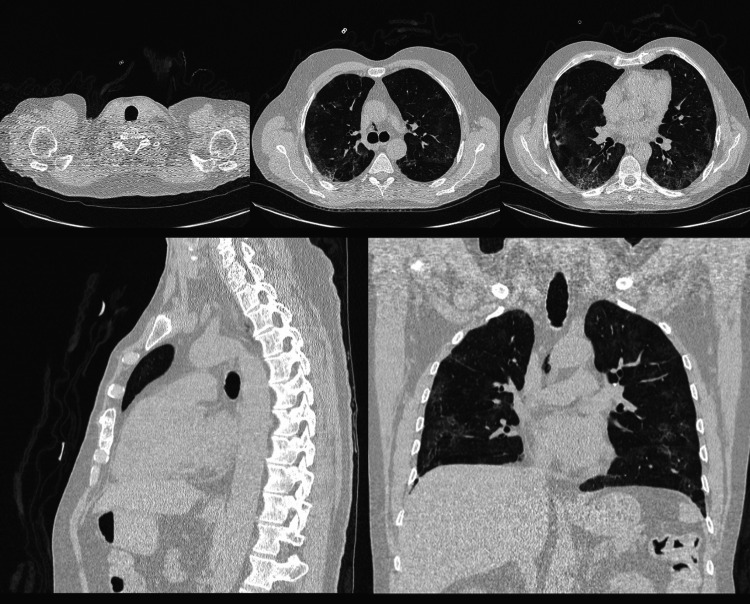
CT scan showing complete resolution of pneumomediastinum and subcutaneous emphysema.

Following conservative management with 100% FiO2 through a non-rebreather mask at night and continued corticosteroid therapy, the patient showed gradual clinical improvement. A follow-up chest CT after seven days revealed resolution of the subcutaneous and mediastinal emphysema, as well as significant improvement in pulmonary lesions (Figure 3).

The patient was discharged on day 27 in stable condition, under corticosteroid therapy with 60 mg prednisolone daily.

## Discussion

This case, occurring at the epicenter of the COVID-19 pneumonia outbreak, illustrates a rare but significant complication of severe COVID-19 pneumonia: pneumomediastinum and subcutaneous emphysema, occurring without invasive mechanical ventilation. Common presenting symptoms include chest pain (60-100%), dyspnea (75%), coughing spells (80%), neck pain (36%), or dysphagia; however, some cases may present without complaints or provoking factors [6]. Although pneumomediastinum is a well-recognized complication typically associated with barotrauma from mechanical ventilation, its occurrence in patients ventilated non-invasively suggests that the pathophysiology in COVID-19 may differ from more typical cases of barotrauma.

The underlying mechanism of pneumomediastinum in COVID-19 patients remains speculative but is thought to result from diffuse alveolar damage. COVID-19, like other viral pneumonias, can lead to acute respiratory distress syndrome (ARDS), characterized by severe lung inflammation, alveolar edema, and diffuse alveolar damage. This damage may increase the vulnerability of alveoli to rupture under pressure, allowing air to escape into the interstitial spaces and migrate into the mediastinum, a phenomenon known as the 'Macklin effect'. In COVID-19, this alveolar rupture is believed to be exacerbated by the virus's impact on lung tissue structure, weakening the alveolar walls and making them more prone to rupture even under modest pressure increases, such as those generated by non-invasive ventilation or spontaneous breathing [7].

Fox SE et al. published a case series of COVID-19 autopsies and found that the dominant pathological process in all cases was diffuse alveolar damage with a mononuclear response around thrombosed small vessels. They suggest that the maladaptive immune response plays a significant role in severe COVID-19 and that the mechanism of air leak observed could be related to significant alveolar damage [3,6,7].

In this case, the patient received HFNC therapy and CPAP, both of which increase alveolar pressure and could potentially contribute to the development of pneumomediastinum. HFNC, while delivering oxygen at high flow rates, can provide positive end-expiratory pressure (PEEP), which may have contributed to alveolar overdistension and rupture. Similarly, CPAP increases airway pressure and could exacerbate air leaks in the context of already damaged alveoli [7].

Pneumomediastinum and subcutaneous emphysema have been reported in patients with COVID-19, both in those receiving mechanical ventilation and those treated with non-invasive ventilation modalities. A systematic review by Quincho-Lopez A et al. identified a growing number of COVID-19 patients with spontaneous pneumomediastinum, many of whom had no history of mechanical ventilation, tobacco use, or underlying lung disease [1]. Similarly, a case report by Sun et al. described a COVID-19 patient who developed pneumomediastinum after treatment with HFNC, further suggesting that the barotrauma associated with non-invasive ventilation in the context of COVID-19 is a likely contributor to this complication [7].

While non-invasive ventilation has been considered a safer alternative to intubation, especially during the COVID-19 pandemic to avoid complications associated with invasive mechanical ventilation, emerging data suggest that even non-invasive modes can lead to barotrauma in this patient population [5,8,9].

This case emphasizes the need for close monitoring of patients receiving HFNC or CPAP, particularly those with worsening respiratory function, as they may be at higher risk for these complications. Once signs of these complications are observed, special attention should be paid, and active measures should be taken. Therefore, early imaging diagnosis and timely treatment of COVID-19 complications can improve therapeutic outcomes and reduce mortality [7].

The management of pneumomediastinum and subcutaneous emphysema in COVID-19 patients remains largely conservative. In most cases, including the one presented here, the condition resolves spontaneously with supportive care. Key aspects of management include close monitoring for signs of progression, bed rest, oxygen therapy, and adequate analgesia. In our patient, pneumomediastinum was identified through imaging following the detection of neck crepitus, highlighting the importance of early radiological investigation when patients present with new physical signs, such as subcutaneous emphysema, or worsening respiratory function [3,8].

Given the spontaneous nature of resolution in most cases, invasive interventions are rarely required. However, clinicians should remain vigilant for potential complications, such as pneumothorax, tension pneumomediastinum, or worsening respiratory failure, which may necessitate more aggressive interventions, including chest tube placement or mechanical ventilation [1].

## Conclusions

This case underscores the importance of heightened awareness of pneumomediastinum and subcutaneous emphysema as potential complications in patients with severe COVID-19, especially those receiving non-invasive ventilation. These conditions should be considered in the differential diagnosis when patients exhibit worsening symptoms.

Further research is necessary to understand the incidence, mechanisms, and risk factors associated with pneumomediastinum in COVID-19 patients, as well as to develop guidelines for the safe use of non-invasive ventilation in this population.
